# 13,13′-Bi(dibenzo[*a*,*i*]fluorenyl­idene)

**DOI:** 10.1107/S2414314622001699

**Published:** 2022-02-17

**Authors:** Dieter Schollmeyer, Heiner Detert

**Affiliations:** a University of Mainz, Department of Chemistry, Duesbergweg 10-14, 55099 Mainz, Germany; Goethe-Universität Frankfurt, Germany

**Keywords:** crystal structure, strain, twisted alkene, polycyclic aromatic, fulvene, twin

## Abstract

The asymmetric unit of the title compound, C_42_H_24_, contains two almost identical mol­ecules. The mol­ecules have approximate *D*_2_ symmetry. They show a largely twisted double bond, the mol­ecular halves enclosing dihedral angles of 62.86 (4) and 61.22 (3)°. The crystal studied was twinned by non-merohedry.

## Abstract 2

The asymmetric unit of the title compound contains two almost identical mol­ecules, which have approximate *D*_2_ symmetry. They show a largely twisted double bond, the mol­ecular halves enclosing dihedral angles of 62.86 (4) and 61.22 (3)°.

## Graphical abstract

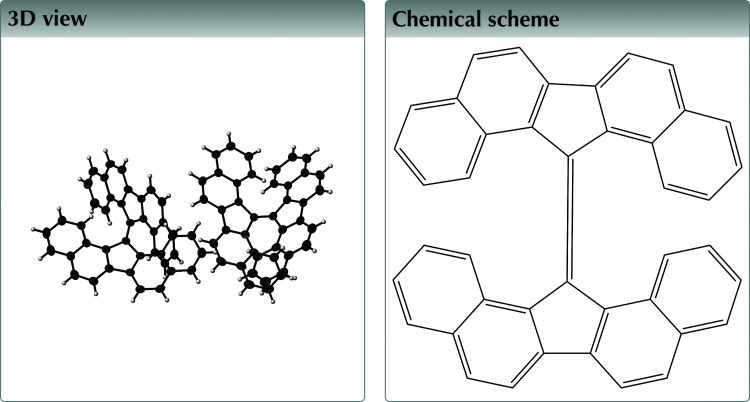

## Structure description

**Figure 1:**
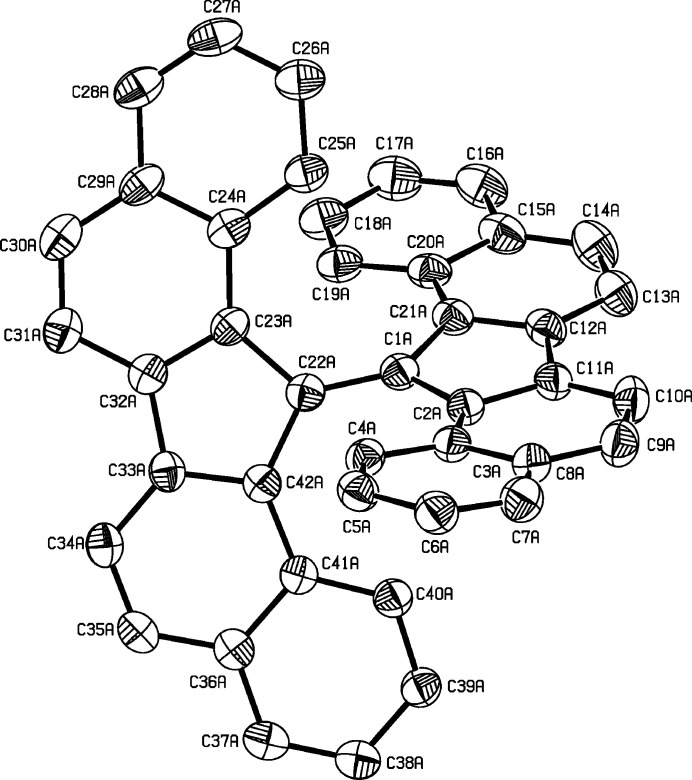
View of one of the two mol­ecules in the asymmetric unit of the title compound. Displacement ellipsoids are drawn at the 50% probability level. H atoms are omitted for clarity.

**Figure 2:**
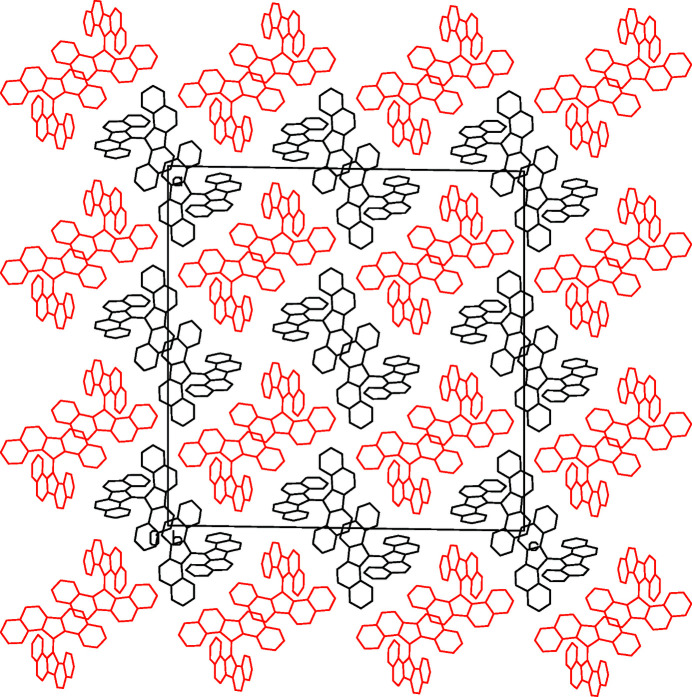
A partial packing diagram viewed along the *b*-axis. The two symmetry-independent mol­ecules are drawn with different colours.

Benzoannulated fulvenes attract chemists because of their electronic properties (Bergmann *et al.* 1953*a* ▸,*b* ▸), their intense colour, the question of aromaticity and the participation of radical character in their ground state (Kanawati *et al.*, 2012 ▸; Wentrup *et al.*, 2016 ▸). In the overcrowded alkene, steric strain prevents the mol­ecule from being planar; a single line in the ESR spectrum (Franzen & Joschek, 1961 ▸) and a simulated torsion angle of 52° (based on diffraction data, Beck *et al.*, 1994 ▸) between the two penta­cyclic halves give evidence for a significant contribution of a radical species. The unit cell is filled with 16 mol­ecules, eight mol­ecules of type *A* (Fig. 1 ▸) and eight of type *B*. The two independent mol­ecules, *A* and *B*, are nearly identical. A packing diagram shows their mutual orientation in the unit cell (Fig. 2 ▸). Mol­ecule *A* consists of two planes, *A*1, *A*2, connected *via* the central C1*A*—C22*A* bond. The maximum deviation of carbon atoms from the mean planes are −0.254 (4) Å for C2 (*A*1) and 0.162 (4) Å for C39 (*A*2). The dihedral angle between these planes is 62.86 (4)° and the length of the central C1*A*—C22*A* bond is 1.408 (5) Å. The values for mol­ecule *B* are very similar: maximum deviations from the mean planes are −0.293 (4) Å for C5 (*B*1) and 0.170 (4) Å for C26 (*B*2). The dihedral angle amounts to 61.22 (3)° and the length of the central C1*B*—C22*B* bond is 1.395 (5) Å. Whereas the length of the twisted and elongated bond connecting the two halves of mol­ecules *A* and *B* [1.408 (5) and 1.395 (5) Å, respectively] correlates very well with the calculated value of 1.408 Å, the calculated torsion angle of 52° is about 10° too small. The solid material gives a very weak single ESR signal.

## Synthesis and crystallization

The dark-green title compound was prepared according to literature procedures (Magidson, 1925 ▸; Detert & Schollmeyer, 2019 ▸). Single crystals of the title compound were obtained by slow evaporation of a saturated solution in chloro­form-*d*_1_.

## Refinement

Crystal data, data collection and structure refinement details are summarized in Table 1 ▸. The crystal was twinned by non-merohedry. The fractional contribution of the minor twin component refined to 0.4648 (15).

## Supplementary Material

Crystal structure: contains datablock(s) I, global. DOI: 10.1107/S2414314622001699/bt4120sup1.cif

Structure factors: contains datablock(s) I. DOI: 10.1107/S2414314622001699/bt4120Isup2.hkl

Supporting information file. DOI: 10.1107/S2414314622001699/bt4120Isup3.cml

CCDC reference: 2151512

Additional supporting information:  crystallographic information; 3D view; checkCIF report

## Figures and Tables

**Table 1:** Experimental details

Crystal data	
Chemical formula	C_42_H_24_
*M*_r_	528.61
Crystal system, space group	Monoclinic, *C*2/*c*
Temperature (K)	120
*a*, *b*, *c* (Å)	36.6150 (18), 8.0709 (3), 36.282 (3)
β (°)	90.863 (5)
*V* (Å^3^)	10720.8 (10)
*Z*	16
Radiation type	Mo *K*α
μ (mm^−1^)	0.07
Crystal size (mm)	0.28 × 0.22 × 0.15

Data collection	
Diffractometer	Stoe IPDS 2T
No. of measured, independent and observed [*I* > 2σ(*I*)] reflections	41460, 41460, 22495
(sin θ/λ)_max_ (Å^−1^)	0.661

Refinement	
*R*[*F* ^2^ > 2σ(*F* ^2^)], *wR*(*F* ^2^), *S*	0.072, 0.228, 1.03
No. of reflections	41460
No. of parameters	758
H-atom treatment	H-atom parameters constrained
Δρ_max_, Δρ_min_ (e Å^−3^)	0.32, −0.33

Computer programs: *X-AREA* *WinXpose*, *Recipe* and *Integrate* (Stoe & Cie, 2019 ▸), *SHELXT2014* (Sheldrick, 2015*a* ▸), *SHELXL2018/3* (Sheldrick, 2015*b* ▸) and *PLATON* (Spek, 2020 ▸).

## full crystallographic data

### Crystal data

**Table:**

C_42_H_24_	*F*(000) = 4416
*M**_r_* = 528.61	*D*_x_ = 1.310 Mg m^−^^3^
Monoclinic, *C*2/*c*	Mo *K*α radiation, λ = 0.71073 Å
*a* = 36.6150 (18) Å	Cell parameters from 16781 reflections
*b* = 8.0709 (3) Å	θ = 2.2–28.4°
*c* = 36.282 (3) Å	µ = 0.07 mm^−^^1^
β = 90.863 (5)°	*T* = 120 K
*V* = 10720.8 (10) Å^3^	Block, green
*Z* = 16	0.28 × 0.22 × 0.15 mm

### Data collection

**Table:**

Stoe IPDS 2T diffractometer	22495 reflections with *I* > 2σ(*I*)
Radiation source: sealed X-ray tube, 12 x 0.4 mm long-fine focus	θ_max_ = 28.0°, θ_min_ = 2.2°
Detector resolution: 6.67 pixels mm^-1^	*h* = −48→48
rotation method scans	*k* = −10→10
41460 measured reflections	*l* = −47→47
41460 independent reflections	

### Refinement

**Table:**

Refinement on *F*^2^	Primary atom site location: dual
Least-squares matrix: full	Hydrogen site location: inferred from neighbouring sites
*R*[*F*^2^ > 2σ(*F*^2^)] = 0.072	H-atom parameters constrained
*wR*(*F*^2^) = 0.228	*w* = 1/[σ^2^(*F*_o_^2^) + (0.0953*P*)^2^ + 14.3038*P*] where *P* = (*F*_o_^2^ + 2*F*_c_^2^)/3
*S* = 1.03	(Δ/σ)_max_ < 0.001
41460 reflections	Δρ_max_ = 0.32 e Å^−^^3^
758 parameters	Δρ_min_ = −0.33 e Å^−^^3^
0 restraints	

### Special details

**Table:**

Geometry. All esds (except the esd in the dihedral angle between two l.s. planes) are estimated using the full covariance matrix. The cell esds are taken into account individually in the estimation of esds in distances, angles and torsion angles; correlations between esds in cell parameters are only used when they are defined by crystal symmetry. An approximate (isotropic) treatment of cell esds is used for estimating esds involving l.s. planes.
Refinement. Hydrogen atoms attached to carbons were placed at calculated positions and were refined in the riding-model approximation with C–H = 0.95 Å, and with *U*_iso_(H) = 1.2 *U*_eq_(C). Refined as a two-component twin.

### Fractional atomic coordinates and isotropic or equivalent isotropic displacement parameters (Å^2^)

**Table:**

	*x*	*y*	*z*	*U*_iso_*/*U*_eq_	
C1A	0.07601 (11)	0.8616 (4)	0.43505 (10)	0.0313 (8)	
C2A	0.04302 (11)	0.8118 (5)	0.45484 (10)	0.0325 (8)	
C3A	0.00551 (11)	0.8345 (5)	0.44590 (11)	0.0330 (9)	
C4A	−0.00823 (11)	0.9106 (5)	0.41312 (11)	0.0338 (9)	
H4A	0.008442	0.946696	0.394983	0.041*	
C5A	−0.04480 (11)	0.9328 (5)	0.40715 (11)	0.0374 (9)	
H5A	−0.053143	0.983644	0.384954	0.045*	
C6A	−0.07020 (12)	0.8817 (5)	0.43333 (11)	0.0423 (10)	
H6A	−0.095587	0.897193	0.428726	0.051*	
C7A	−0.05844 (12)	0.8094 (6)	0.46557 (12)	0.0438 (10)	
H7A	−0.075778	0.773964	0.483114	0.053*	
C8A	−0.02062 (13)	0.7872 (6)	0.47296 (13)	0.0389 (10)	
C9A	−0.00861 (14)	0.7251 (6)	0.50747 (15)	0.0435 (11)	
H9A	−0.026170	0.687692	0.524591	0.052*	
C10A	0.02781 (13)	0.7175 (6)	0.51687 (14)	0.0422 (11)	
H10A	0.035523	0.681950	0.540707	0.051*	
C11A	0.05324 (14)	0.7636 (4)	0.49039 (14)	0.0340 (11)	
C12A	0.09275 (12)	0.7830 (5)	0.49452 (13)	0.0352 (9)	
C13A	0.11511 (14)	0.7405 (5)	0.52453 (16)	0.0422 (13)	
H13A	0.104815	0.693354	0.545993	0.051*	
C14A	0.15180 (15)	0.7670 (6)	0.52286 (16)	0.0493 (14)	
H14A	0.166905	0.740684	0.543561	0.059*	
C15A	0.16780 (12)	0.8335 (5)	0.49064 (12)	0.0423 (10)	
C16A	0.20604 (12)	0.8565 (6)	0.48900 (13)	0.0488 (11)	
H16A	0.220534	0.834888	0.510395	0.059*	
C17A	0.22268 (13)	0.9083 (6)	0.45803 (13)	0.0512 (12)	
H17A	0.248412	0.923200	0.457738	0.061*	
C18A	0.20144 (12)	0.9397 (5)	0.42645 (13)	0.0435 (10)	
H18A	0.213005	0.973170	0.404404	0.052*	
C19A	0.16396 (11)	0.9228 (5)	0.42680 (12)	0.0369 (9)	
H19A	0.150161	0.944754	0.404961	0.044*	
C20A	0.14565 (11)	0.8731 (5)	0.45915 (11)	0.0349 (9)	
C21A	0.10666 (11)	0.8519 (5)	0.46200 (11)	0.0334 (9)	
C22A	0.07728 (10)	0.9001 (4)	0.39723 (10)	0.0302 (8)	
C23A	0.09461 (10)	1.0466 (4)	0.38085 (10)	0.0310 (8)	
C24A	0.10843 (10)	1.1956 (5)	0.39726 (11)	0.0331 (8)	
C25A	0.10835 (13)	1.2314 (5)	0.43535 (15)	0.0387 (11)	
H25A	0.097616	1.154631	0.451812	0.046*	
C26A	0.12339 (12)	1.3741 (5)	0.44906 (13)	0.0449 (11)	
H26A	0.122942	1.395994	0.474774	0.054*	
C27A	0.13953 (12)	1.4885 (5)	0.42501 (13)	0.0455 (11)	
H27A	0.151275	1.584411	0.434692	0.055*	
C28A	0.13843 (11)	1.4630 (5)	0.38838 (13)	0.0431 (10)	
H28A	0.148516	1.544024	0.372508	0.052*	
C29A	0.12261 (11)	1.3183 (5)	0.37287 (12)	0.0374 (9)	
C30A	0.12101 (12)	1.2941 (6)	0.33423 (13)	0.0421 (10)	
H30A	0.131549	1.374603	0.318528	0.051*	
C31A	0.10469 (12)	1.1580 (5)	0.31904 (12)	0.0389 (9)	
H31A	0.102728	1.145550	0.293049	0.047*	
C32A	0.09086 (10)	1.0366 (4)	0.34277 (10)	0.0318 (8)	
C33A	0.07080 (10)	0.8859 (5)	0.33337 (10)	0.0322 (8)	
C34A	0.06220 (11)	0.8233 (5)	0.29800 (11)	0.0357 (9)	
H34A	0.067641	0.885220	0.276506	0.043*	
C35A	0.04593 (11)	0.6719 (5)	0.29539 (11)	0.0381 (10)	
H35A	0.039212	0.629849	0.271794	0.046*	
C36A	0.03880 (10)	0.5757 (5)	0.32729 (11)	0.0335 (9)	
C37A	0.02452 (11)	0.4132 (5)	0.32325 (12)	0.0371 (9)	
H37A	0.018847	0.372400	0.299279	0.044*	
C38A	0.01875 (11)	0.3139 (5)	0.35321 (11)	0.0373 (9)	
H38A	0.009136	0.205395	0.350075	0.045*	
C39A	0.02721 (11)	0.3744 (5)	0.38863 (11)	0.0350 (9)	
H39A	0.023255	0.305642	0.409441	0.042*	
C40A	0.04107 (10)	0.5307 (5)	0.39376 (11)	0.0338 (9)	
H40A	0.046770	0.567463	0.418042	0.041*	
C41A	0.04708 (10)	0.6384 (5)	0.36367 (10)	0.0301 (8)	
C42A	0.06203 (11)	0.8035 (5)	0.36603 (11)	0.0314 (8)	
C1B	0.17996 (10)	0.8223 (4)	0.17836 (10)	0.0298 (8)	
C2B	0.20174 (10)	0.8753 (4)	0.21063 (10)	0.0311 (8)	
C3B	0.19396 (11)	0.8642 (4)	0.24910 (11)	0.0322 (8)	
C4B	0.16185 (11)	0.7937 (5)	0.26409 (11)	0.0324 (8)	
H4B	0.143157	0.754534	0.247878	0.039*	
C5B	0.15727 (12)	0.7810 (5)	0.30123 (13)	0.0369 (9)	
H5B	0.135840	0.730476	0.310470	0.044*	
C6B	0.18403 (12)	0.8422 (6)	0.32604 (12)	0.0451 (11)	
H6B	0.180410	0.835466	0.351873	0.054*	
C7B	0.21519 (12)	0.9113 (5)	0.31268 (11)	0.0431 (10)	
H7B	0.232905	0.954691	0.329479	0.052*	
C8B	0.22172 (11)	0.9199 (5)	0.27428 (11)	0.0367 (9)	
C9B	0.25598 (11)	0.9744 (5)	0.26096 (13)	0.0423 (10)	
H9B	0.273530	1.018062	0.277884	0.051*	
C10B	0.26429 (12)	0.9656 (5)	0.22457 (12)	0.0405 (10)	
H10B	0.287880	0.995326	0.216246	0.049*	
C11B	0.23700 (10)	0.9112 (5)	0.19954 (11)	0.0341 (9)	
C12B	0.23937 (11)	0.8775 (5)	0.16018 (11)	0.0357 (9)	
C13B	0.26945 (12)	0.8977 (6)	0.13727 (12)	0.0454 (11)	
H13B	0.292030	0.936751	0.147151	0.055*	
C14B	0.26618 (12)	0.8611 (6)	0.10081 (12)	0.0496 (11)	
H14B	0.286915	0.869224	0.085488	0.060*	
C15B	0.23212 (12)	0.8108 (6)	0.08541 (11)	0.0399 (9)	
C16B	0.22857 (14)	0.7850 (7)	0.04678 (15)	0.0495 (12)	
H16B	0.249583	0.794073	0.031861	0.059*	
C17B	0.19579 (16)	0.7476 (5)	0.03061 (17)	0.0488 (15)	
H17B	0.193900	0.729870	0.004769	0.059*	
C18B	0.16495 (14)	0.7359 (5)	0.05275 (14)	0.0396 (12)	
H18B	0.141942	0.711281	0.041605	0.047*	
C19B	0.16715 (13)	0.7589 (4)	0.09006 (15)	0.0337 (11)	
H19B	0.145590	0.751305	0.104200	0.040*	
C20B	0.20063 (11)	0.7937 (5)	0.10798 (11)	0.0321 (8)	
C21B	0.20568 (10)	0.8187 (5)	0.14677 (10)	0.0315 (8)	
C22B	0.14263 (11)	0.7877 (5)	0.17778 (11)	0.0297 (8)	
C23B	0.12514 (10)	0.6437 (4)	0.16011 (10)	0.0296 (8)	
C24B	0.14033 (11)	0.4913 (4)	0.14716 (10)	0.0318 (8)	
C25B	0.17781 (11)	0.4493 (5)	0.14893 (11)	0.0354 (9)	
H25B	0.194672	0.525269	0.159703	0.042*	
C26B	0.19051 (13)	0.3003 (5)	0.13535 (12)	0.0412 (10)	
H26B	0.215776	0.274240	0.137022	0.049*	
C27B	0.16596 (13)	0.1882 (5)	0.11915 (12)	0.0430 (10)	
H27B	0.174705	0.088286	0.108630	0.052*	
C28B	0.12987 (14)	0.2219 (5)	0.11844 (13)	0.0401 (11)	
H28B	0.113519	0.142059	0.108303	0.048*	
C29B	0.11555 (11)	0.3713 (5)	0.13220 (11)	0.0351 (9)	
C30B	0.07747 (11)	0.4026 (5)	0.13225 (11)	0.0379 (9)	
H30B	0.061341	0.323963	0.121283	0.045*	
C31B	0.06338 (11)	0.5417 (5)	0.14752 (11)	0.0371 (9)	
H31B	0.037709	0.558316	0.148100	0.044*	
C32B	0.08730 (10)	0.6603 (5)	0.16241 (11)	0.0320 (8)	
C33B	0.07942 (10)	0.8130 (5)	0.18178 (10)	0.0310 (8)	
C34B	0.04491 (11)	0.8804 (5)	0.18937 (11)	0.0355 (9)	
H34B	0.023239	0.821254	0.183182	0.043*	
C35B	0.04314 (11)	1.0326 (5)	0.20582 (11)	0.0370 (9)	
H35B	0.020007	1.078000	0.211702	0.044*	
C36B	0.07533 (10)	1.1238 (5)	0.21423 (10)	0.0330 (9)	
C37B	0.07272 (12)	1.2866 (6)	0.22848 (12)	0.0376 (9)	
H37B	0.049240	1.331869	0.232900	0.045*	
C38B	0.10282 (12)	1.3802 (5)	0.23602 (11)	0.0391 (10)	
H38B	0.100481	1.488176	0.246132	0.047*	
C39B	0.13736 (12)	1.3143 (5)	0.22860 (11)	0.0365 (10)	
H39B	0.158444	1.380073	0.233231	0.044*	
C40B	0.14149 (11)	1.1578 (5)	0.21489 (10)	0.0327 (9)	
H40B	0.165327	1.116781	0.210367	0.039*	
C41B	0.11080 (10)	1.0557 (4)	0.20732 (10)	0.0306 (8)	
C42B	0.11221 (10)	0.8906 (4)	0.19212 (10)	0.0294 (8)	

### Atomic displacement parameters (Å^2^)

**Table:**

	*U*^11^	*U*^22^	*U*^33^	*U*^12^	*U*^13^	*U*^23^
C1A	0.036 (2)	0.0251 (19)	0.033 (2)	0.0041 (16)	−0.0001 (15)	−0.0019 (15)
C2A	0.039 (2)	0.028 (2)	0.030 (2)	0.0011 (18)	−0.0001 (16)	−0.0007 (16)
C3A	0.039 (2)	0.028 (2)	0.032 (2)	−0.0004 (17)	0.0031 (16)	−0.0023 (16)
C4A	0.037 (2)	0.029 (2)	0.036 (2)	0.0008 (17)	0.0037 (16)	0.0002 (16)
C5A	0.041 (2)	0.036 (2)	0.035 (2)	0.0030 (18)	−0.0017 (17)	−0.0025 (17)
C6A	0.040 (2)	0.046 (3)	0.041 (2)	−0.003 (2)	0.0012 (18)	−0.0014 (19)
C7A	0.040 (3)	0.048 (3)	0.043 (3)	−0.003 (2)	0.0061 (18)	0.001 (2)
C8A	0.047 (3)	0.037 (2)	0.033 (2)	−0.005 (2)	0.0030 (18)	0.000 (2)
C9A	0.044 (3)	0.048 (3)	0.038 (3)	−0.004 (2)	0.005 (2)	0.007 (2)
C10A	0.051 (3)	0.043 (2)	0.033 (3)	0.004 (2)	0.001 (2)	0.009 (2)
C11A	0.041 (3)	0.030 (2)	0.031 (3)	0.0014 (16)	0.0004 (19)	−0.0013 (14)
C12A	0.041 (2)	0.0323 (18)	0.032 (2)	0.005 (2)	−0.0043 (17)	−0.0042 (19)
C13A	0.041 (3)	0.049 (3)	0.036 (3)	0.0051 (18)	−0.007 (2)	0.0016 (16)
C14A	0.046 (3)	0.063 (3)	0.039 (3)	0.011 (2)	−0.010 (2)	−0.001 (2)
C15A	0.042 (3)	0.043 (3)	0.042 (3)	0.004 (2)	−0.0092 (18)	−0.007 (2)
C16A	0.040 (3)	0.057 (3)	0.049 (3)	0.002 (2)	−0.012 (2)	−0.009 (2)
C17A	0.041 (3)	0.053 (3)	0.059 (3)	−0.001 (2)	−0.008 (2)	−0.007 (2)
C18A	0.039 (2)	0.037 (2)	0.054 (3)	0.0005 (19)	−0.0004 (19)	−0.002 (2)
C19A	0.036 (2)	0.028 (2)	0.046 (2)	0.0024 (17)	−0.0037 (17)	−0.0034 (17)
C20A	0.038 (2)	0.026 (2)	0.040 (2)	0.0033 (17)	−0.0064 (17)	−0.0051 (16)
C21A	0.038 (2)	0.0269 (19)	0.035 (2)	0.0049 (17)	−0.0035 (16)	−0.0034 (16)
C22A	0.030 (2)	0.0266 (19)	0.034 (2)	0.0052 (16)	0.0002 (15)	−0.0006 (15)
C23A	0.030 (2)	0.0267 (19)	0.037 (2)	0.0028 (16)	0.0020 (15)	0.0022 (16)
C24A	0.027 (2)	0.028 (2)	0.044 (2)	0.0046 (17)	0.0003 (16)	0.0016 (19)
C25A	0.045 (3)	0.028 (2)	0.043 (3)	0.0016 (18)	0.001 (2)	−0.0016 (17)
C26A	0.049 (3)	0.032 (2)	0.053 (3)	0.004 (2)	0.001 (2)	−0.007 (2)
C27A	0.043 (3)	0.026 (2)	0.068 (3)	0.0009 (19)	0.003 (2)	−0.008 (2)
C28A	0.039 (2)	0.029 (2)	0.062 (3)	0.0003 (18)	0.007 (2)	0.000 (2)
C29A	0.034 (2)	0.025 (2)	0.053 (3)	0.0035 (17)	0.0049 (18)	0.0043 (18)
C30A	0.039 (2)	0.036 (2)	0.051 (3)	−0.001 (2)	0.0085 (19)	0.009 (2)
C31A	0.041 (2)	0.037 (2)	0.039 (2)	0.0028 (19)	0.0040 (18)	0.0064 (18)
C32A	0.028 (2)	0.031 (2)	0.036 (2)	0.0055 (16)	0.0000 (15)	0.0022 (16)
C33A	0.033 (2)	0.034 (2)	0.030 (2)	0.0033 (16)	0.0006 (15)	0.0023 (16)
C34A	0.033 (2)	0.041 (3)	0.032 (2)	0.0038 (18)	0.0014 (16)	0.0044 (18)
C35A	0.038 (2)	0.045 (3)	0.031 (2)	0.0014 (19)	−0.0019 (16)	−0.0045 (18)
C36A	0.028 (2)	0.037 (2)	0.035 (2)	0.0047 (17)	−0.0014 (15)	−0.0002 (17)
C37A	0.031 (2)	0.039 (2)	0.041 (2)	0.0011 (18)	−0.0004 (16)	−0.0080 (18)
C38A	0.034 (2)	0.036 (2)	0.042 (2)	0.0017 (18)	−0.0025 (17)	−0.0052 (19)
C39A	0.039 (2)	0.029 (2)	0.037 (2)	0.0036 (17)	0.0004 (16)	0.0011 (16)
C40A	0.036 (2)	0.032 (2)	0.034 (2)	0.0008 (17)	0.0000 (16)	−0.0005 (16)
C41A	0.027 (2)	0.032 (2)	0.032 (2)	0.0014 (16)	0.0019 (14)	0.0000 (16)
C42A	0.030 (2)	0.032 (2)	0.032 (2)	0.0018 (18)	−0.0006 (15)	0.0001 (18)
C1B	0.032 (2)	0.0222 (19)	0.035 (2)	0.0009 (15)	0.0015 (15)	0.0010 (15)
C2B	0.030 (2)	0.0249 (19)	0.039 (2)	−0.0010 (15)	−0.0014 (15)	−0.0024 (16)
C3B	0.034 (2)	0.0261 (19)	0.036 (2)	0.0015 (16)	−0.0020 (15)	−0.0012 (16)
C4B	0.031 (2)	0.0331 (19)	0.033 (2)	0.0035 (18)	−0.0018 (15)	0.0014 (19)
C5B	0.031 (2)	0.044 (2)	0.036 (3)	0.0046 (19)	0.0002 (17)	0.004 (2)
C6B	0.039 (2)	0.061 (3)	0.035 (2)	0.005 (2)	−0.0004 (17)	−0.001 (2)
C7B	0.042 (3)	0.051 (3)	0.037 (2)	−0.002 (2)	−0.0060 (17)	−0.0061 (19)
C8B	0.033 (2)	0.036 (2)	0.041 (2)	0.0009 (18)	−0.0036 (16)	−0.0034 (18)
C9B	0.038 (3)	0.043 (2)	0.045 (2)	−0.007 (2)	−0.0068 (19)	−0.007 (2)
C10B	0.033 (2)	0.045 (3)	0.043 (3)	−0.0077 (19)	−0.0020 (18)	−0.0002 (19)
C11B	0.030 (2)	0.032 (2)	0.040 (2)	−0.0018 (16)	−0.0005 (16)	0.0023 (17)
C12B	0.033 (2)	0.034 (2)	0.040 (2)	0.0000 (17)	0.0020 (16)	0.0007 (17)
C13B	0.031 (2)	0.059 (3)	0.046 (3)	−0.007 (2)	0.0040 (18)	−0.004 (2)
C14B	0.036 (2)	0.071 (3)	0.043 (3)	−0.001 (2)	0.0096 (19)	−0.002 (2)
C15B	0.036 (2)	0.045 (3)	0.038 (2)	0.001 (2)	0.0060 (17)	−0.004 (2)
C16B	0.039 (3)	0.072 (3)	0.038 (3)	0.004 (2)	0.009 (2)	−0.002 (3)
C17B	0.049 (3)	0.064 (4)	0.034 (3)	0.007 (2)	0.006 (2)	−0.0061 (17)
C18B	0.039 (3)	0.044 (3)	0.036 (3)	0.0048 (17)	−0.001 (2)	−0.0024 (16)
C19B	0.033 (2)	0.031 (3)	0.037 (3)	0.0017 (14)	0.0024 (19)	0.0000 (14)
C20B	0.032 (2)	0.0275 (18)	0.037 (2)	0.0039 (18)	0.0053 (16)	0.0008 (19)
C21B	0.032 (2)	0.025 (2)	0.037 (2)	0.0022 (16)	0.0031 (15)	0.0009 (16)
C22B	0.032 (2)	0.0235 (16)	0.034 (2)	−0.0010 (18)	−0.0008 (15)	0.0021 (18)
C23B	0.030 (2)	0.0275 (19)	0.031 (2)	−0.0007 (16)	−0.0041 (14)	0.0022 (15)
C24B	0.036 (2)	0.0281 (19)	0.031 (2)	−0.0024 (16)	−0.0008 (15)	0.0021 (16)
C25B	0.043 (2)	0.026 (2)	0.037 (2)	0.0004 (17)	0.0005 (17)	0.0013 (16)
C26B	0.048 (3)	0.033 (2)	0.043 (3)	0.006 (2)	0.0010 (19)	0.004 (2)
C27B	0.064 (3)	0.025 (2)	0.039 (3)	0.004 (2)	0.001 (2)	−0.0027 (18)
C28B	0.054 (3)	0.0267 (19)	0.040 (3)	−0.005 (2)	−0.006 (2)	0.000 (2)
C29B	0.045 (2)	0.028 (2)	0.032 (2)	−0.0053 (18)	−0.0040 (16)	0.0004 (16)
C30B	0.039 (2)	0.033 (2)	0.041 (2)	−0.0069 (18)	−0.0069 (17)	−0.0016 (17)
C31B	0.038 (2)	0.036 (2)	0.037 (2)	−0.0041 (18)	−0.0033 (17)	0.0011 (17)
C32B	0.032 (2)	0.0288 (19)	0.036 (2)	−0.0030 (16)	−0.0013 (15)	0.0049 (17)
C33B	0.029 (2)	0.033 (2)	0.032 (2)	−0.0004 (17)	−0.0015 (15)	0.0004 (17)
C34B	0.029 (2)	0.038 (2)	0.040 (2)	−0.0019 (17)	0.0004 (16)	0.0029 (17)
C35B	0.035 (2)	0.043 (2)	0.034 (2)	0.0047 (18)	0.0005 (16)	0.0031 (18)
C36B	0.028 (2)	0.038 (2)	0.034 (2)	0.0028 (17)	0.0018 (15)	0.0051 (17)
C37B	0.042 (2)	0.0347 (19)	0.036 (3)	0.006 (2)	0.0030 (18)	−0.001 (2)
C38B	0.050 (3)	0.034 (2)	0.033 (2)	0.0053 (19)	0.0030 (18)	0.0013 (17)
C39B	0.044 (2)	0.031 (2)	0.034 (2)	−0.0016 (19)	0.0026 (17)	0.0026 (18)
C40B	0.034 (2)	0.032 (2)	0.032 (2)	0.0001 (17)	−0.0006 (15)	0.0027 (17)
C41B	0.035 (2)	0.0284 (19)	0.029 (2)	0.0026 (16)	0.0004 (15)	0.0036 (15)
C42B	0.0271 (19)	0.031 (2)	0.030 (2)	0.0012 (16)	−0.0006 (14)	0.0031 (15)

### Geometric parameters (Å, º)

**Table:**

C1A—C22A	1.408 (5)	C1B—C22B	1.395 (5)
C1A—C2A	1.471 (5)	C1B—C2B	1.470 (5)
C1A—C21A	1.479 (5)	C1B—C21B	1.494 (5)
C2A—C11A	1.393 (6)	C2B—C11B	1.389 (5)
C2A—C3A	1.418 (5)	C2B—C3B	1.432 (5)
C3A—C4A	1.424 (5)	C3B—C4B	1.421 (5)
C3A—C8A	1.433 (6)	C3B—C8B	1.429 (5)
C4A—C5A	1.365 (6)	C4B—C5B	1.364 (6)
C4A—H4A	0.9500	C4B—H4B	0.9500
C5A—C6A	1.401 (5)	C5B—C6B	1.410 (6)
C5A—H5A	0.9500	C5B—H5B	0.9500
C6A—C7A	1.371 (6)	C6B—C7B	1.365 (6)
C6A—H6A	0.9500	C6B—H6B	0.9500
C7A—C8A	1.418 (6)	C7B—C8B	1.419 (6)
C7A—H7A	0.9500	C7B—H7B	0.9500
C8A—C9A	1.413 (7)	C8B—C9B	1.421 (5)
C9A—C10A	1.373 (7)	C9B—C10B	1.361 (6)
C9A—H9A	0.9500	C9B—H9B	0.9500
C10A—C11A	1.398 (6)	C10B—C11B	1.410 (5)
C10A—H10A	0.9500	C10B—H10B	0.9500
C11A—C12A	1.461 (6)	C11B—C12B	1.457 (5)
C12A—C13A	1.395 (6)	C12B—C13B	1.399 (5)
C12A—C21A	1.406 (6)	C12B—C21B	1.402 (5)
C13A—C14A	1.363 (7)	C13B—C14B	1.359 (6)
C13A—H13A	0.9500	C13B—H13B	0.9500
C14A—C15A	1.421 (7)	C14B—C15B	1.418 (6)
C14A—H14A	0.9500	C14B—H14B	0.9500
C15A—C16A	1.414 (6)	C15B—C16B	1.421 (7)
C15A—C20A	1.428 (5)	C15B—C20B	1.431 (5)
C16A—C17A	1.353 (6)	C16B—C17B	1.362 (8)
C16A—H16A	0.9500	C16B—H16B	0.9500
C17A—C18A	1.398 (6)	C17B—C18B	1.399 (7)
C17A—H17A	0.9500	C17B—H17B	0.9500
C18A—C19A	1.379 (6)	C18B—C19B	1.368 (7)
C18A—H18A	0.9500	C18B—H18B	0.9500
C19A—C20A	1.418 (6)	C19B—C20B	1.407 (6)
C19A—H19A	0.9500	C19B—H19B	0.9500
C20A—C21A	1.443 (5)	C20B—C21B	1.431 (5)
C22A—C23A	1.471 (5)	C22B—C23B	1.470 (5)
C22A—C42A	1.477 (5)	C22B—C42B	1.489 (5)
C23A—C32A	1.389 (5)	C23B—C32B	1.396 (5)
C23A—C24A	1.431 (5)	C23B—C24B	1.432 (5)
C24A—C25A	1.412 (6)	C24B—C25B	1.414 (5)
C24A—C29A	1.431 (6)	C24B—C29B	1.429 (5)
C25A—C26A	1.367 (6)	C25B—C26B	1.382 (6)
C25A—H25A	0.9500	C25B—H25B	0.9500
C26A—C27A	1.407 (6)	C26B—C27B	1.399 (6)
C26A—H26A	0.9500	C26B—H26B	0.9500
C27A—C28A	1.345 (6)	C27B—C28B	1.349 (7)
C27A—H27A	0.9500	C27B—H27B	0.9500
C28A—C29A	1.416 (6)	C28B—C29B	1.409 (6)
C28A—H28A	0.9500	C28B—H28B	0.9500
C29A—C30A	1.416 (6)	C29B—C30B	1.417 (6)
C30A—C31A	1.363 (6)	C30B—C31B	1.357 (5)
C30A—H30A	0.9500	C30B—H30B	0.9500
C31A—C32A	1.404 (5)	C31B—C32B	1.400 (5)
C31A—H31A	0.9500	C31B—H31B	0.9500
C32A—C33A	1.458 (5)	C32B—C33B	1.450 (6)
C33A—C42A	1.400 (5)	C33B—C42B	1.400 (5)
C33A—C34A	1.410 (5)	C33B—C34B	1.407 (5)
C34A—C35A	1.362 (6)	C34B—C35B	1.368 (5)
C34A—H34A	0.9500	C34B—H34B	0.9500
C35A—C36A	1.421 (5)	C35B—C36B	1.419 (5)
C35A—H35A	0.9500	C35B—H35B	0.9500
C36A—C37A	1.419 (5)	C36B—C37B	1.416 (6)
C36A—C41A	1.442 (5)	C36B—C41B	1.436 (5)
C37A—C38A	1.369 (6)	C37B—C38B	1.361 (6)
C37A—H37A	0.9500	C37B—H37B	0.9500
C38A—C39A	1.405 (5)	C38B—C39B	1.402 (6)
C38A—H38A	0.9500	C38B—H38B	0.9500
C39A—C40A	1.371 (5)	C39B—C40B	1.366 (6)
C39A—H39A	0.9500	C39B—H39B	0.9500
C40A—C41A	1.415 (5)	C40B—C41B	1.417 (5)
C40A—H40A	0.9500	C40B—H40B	0.9500
C41A—C42A	1.443 (5)	C41B—C42B	1.444 (5)
			
C22A—C1A—C2A	125.1 (4)	C22B—C1B—C2B	126.1 (3)
C22A—C1A—C21A	128.3 (4)	C22B—C1B—C21B	127.8 (4)
C2A—C1A—C21A	106.5 (3)	C2B—C1B—C21B	106.0 (3)
C11A—C2A—C3A	119.6 (4)	C11B—C2B—C3B	119.7 (4)
C11A—C2A—C1A	108.5 (4)	C11B—C2B—C1B	109.0 (3)
C3A—C2A—C1A	130.8 (4)	C3B—C2B—C1B	130.1 (3)
C2A—C3A—C4A	125.1 (4)	C4B—C3B—C8B	117.8 (4)
C2A—C3A—C8A	117.6 (4)	C4B—C3B—C2B	125.2 (4)
C4A—C3A—C8A	117.2 (4)	C8B—C3B—C2B	117.0 (4)
C5A—C4A—C3A	121.5 (4)	C5B—C4B—C3B	121.5 (4)
C5A—C4A—H4A	119.2	C5B—C4B—H4B	119.2
C3A—C4A—H4A	119.2	C3B—C4B—H4B	119.2
C4A—C5A—C6A	120.9 (4)	C4B—C5B—C6B	120.6 (4)
C4A—C5A—H5A	119.6	C4B—C5B—H5B	119.7
C6A—C5A—H5A	119.6	C6B—C5B—H5B	119.7
C7A—C6A—C5A	120.0 (4)	C7B—C6B—C5B	119.5 (4)
C7A—C6A—H6A	120.0	C7B—C6B—H6B	120.2
C5A—C6A—H6A	120.0	C5B—C6B—H6B	120.2
C6A—C7A—C8A	120.6 (4)	C6B—C7B—C8B	121.5 (4)
C6A—C7A—H7A	119.7	C6B—C7B—H7B	119.2
C8A—C7A—H7A	119.7	C8B—C7B—H7B	119.2
C9A—C8A—C7A	120.2 (4)	C7B—C8B—C9B	120.8 (4)
C9A—C8A—C3A	120.0 (4)	C7B—C8B—C3B	118.9 (4)
C7A—C8A—C3A	119.8 (4)	C9B—C8B—C3B	120.2 (4)
C10A—C9A—C8A	121.6 (5)	C10B—C9B—C8B	121.7 (4)
C10A—C9A—H9A	119.2	C10B—C9B—H9B	119.2
C8A—C9A—H9A	119.2	C8B—C9B—H9B	119.2
C9A—C10A—C11A	118.1 (5)	C9B—C10B—C11B	118.3 (4)
C9A—C10A—H10A	120.9	C9B—C10B—H10B	120.8
C11A—C10A—H10A	120.9	C11B—C10B—H10B	120.8
C2A—C11A—C10A	122.6 (4)	C2B—C11B—C10B	122.1 (4)
C2A—C11A—C12A	108.5 (4)	C2B—C11B—C12B	108.3 (3)
C10A—C11A—C12A	128.8 (5)	C10B—C11B—C12B	129.6 (4)
C13A—C12A—C21A	122.4 (4)	C13B—C12B—C21B	122.0 (4)
C13A—C12A—C11A	128.5 (4)	C13B—C12B—C11B	128.3 (4)
C21A—C12A—C11A	109.1 (4)	C21B—C12B—C11B	109.8 (3)
C14A—C13A—C12A	119.6 (5)	C14B—C13B—C12B	119.6 (4)
C14A—C13A—H13A	120.2	C14B—C13B—H13B	120.2
C12A—C13A—H13A	120.2	C12B—C13B—H13B	120.2
C13A—C14A—C15A	121.0 (5)	C13B—C14B—C15B	120.7 (4)
C13A—C14A—H14A	119.5	C13B—C14B—H14B	119.7
C15A—C14A—H14A	119.5	C15B—C14B—H14B	119.7
C16A—C15A—C14A	120.3 (4)	C14B—C15B—C16B	119.8 (4)
C16A—C15A—C20A	119.2 (4)	C14B—C15B—C20B	120.9 (4)
C14A—C15A—C20A	120.4 (4)	C16B—C15B—C20B	119.2 (4)
C17A—C16A—C15A	122.3 (4)	C17B—C16B—C15B	121.6 (5)
C17A—C16A—H16A	118.9	C17B—C16B—H16B	119.2
C15A—C16A—H16A	118.9	C15B—C16B—H16B	119.2
C16A—C17A—C18A	119.0 (4)	C16B—C17B—C18B	118.8 (5)
C16A—C17A—H17A	120.5	C16B—C17B—H17B	120.6
C18A—C17A—H17A	120.5	C18B—C17B—H17B	120.6
C19A—C18A—C17A	121.1 (4)	C19B—C18B—C17B	121.5 (5)
C19A—C18A—H18A	119.5	C19B—C18B—H18B	119.2
C17A—C18A—H18A	119.5	C17B—C18B—H18B	119.2
C18A—C19A—C20A	121.2 (4)	C18B—C19B—C20B	121.5 (5)
C18A—C19A—H19A	119.4	C18B—C19B—H19B	119.3
C20A—C19A—H19A	119.4	C20B—C19B—H19B	119.3
C19A—C20A—C15A	117.1 (4)	C19B—C20B—C15B	117.3 (4)
C19A—C20A—C21A	125.0 (4)	C19B—C20B—C21B	125.5 (4)
C15A—C20A—C21A	117.8 (4)	C15B—C20B—C21B	117.1 (4)
C12A—C21A—C20A	118.6 (4)	C12B—C21B—C20B	119.3 (4)
C12A—C21A—C1A	107.2 (4)	C12B—C21B—C1B	106.8 (3)
C20A—C21A—C1A	133.4 (4)	C20B—C21B—C1B	133.1 (4)
C1A—C22A—C23A	126.3 (3)	C1B—C22B—C23B	125.8 (4)
C1A—C22A—C42A	127.8 (3)	C1B—C22B—C42B	128.4 (4)
C23A—C22A—C42A	105.9 (3)	C23B—C22B—C42B	105.7 (3)
C32A—C23A—C24A	119.5 (3)	C32B—C23B—C24B	119.5 (3)
C32A—C23A—C22A	108.6 (3)	C32B—C23B—C22B	108.9 (3)
C24A—C23A—C22A	131.2 (3)	C24B—C23B—C22B	130.8 (3)
C25A—C24A—C29A	118.0 (4)	C25B—C24B—C29B	117.7 (3)
C25A—C24A—C23A	125.0 (4)	C25B—C24B—C23B	124.9 (3)
C29A—C24A—C23A	117.0 (4)	C29B—C24B—C23B	117.3 (3)
C26A—C25A—C24A	121.5 (5)	C26B—C25B—C24B	121.6 (4)
C26A—C25A—H25A	119.3	C26B—C25B—H25B	119.2
C24A—C25A—H25A	119.3	C24B—C25B—H25B	119.2
C25A—C26A—C27A	119.9 (4)	C25B—C26B—C27B	119.7 (4)
C25A—C26A—H26A	120.1	C25B—C26B—H26B	120.2
C27A—C26A—H26A	120.1	C27B—C26B—H26B	120.2
C28A—C27A—C26A	120.4 (4)	C28B—C27B—C26B	120.0 (4)
C28A—C27A—H27A	119.8	C28B—C27B—H27B	120.0
C26A—C27A—H27A	119.8	C26B—C27B—H27B	120.0
C27A—C28A—C29A	121.7 (4)	C27B—C28B—C29B	122.4 (4)
C27A—C28A—H28A	119.2	C27B—C28B—H28B	118.8
C29A—C28A—H28A	119.2	C29B—C28B—H28B	118.8
C30A—C29A—C28A	121.2 (4)	C28B—C29B—C30B	121.6 (4)
C30A—C29A—C24A	120.4 (4)	C28B—C29B—C24B	118.5 (4)
C28A—C29A—C24A	118.4 (4)	C30B—C29B—C24B	119.9 (4)
C31A—C30A—C29A	121.5 (4)	C31B—C30B—C29B	121.9 (4)
C31A—C30A—H30A	119.2	C31B—C30B—H30B	119.1
C29A—C30A—H30A	119.2	C29B—C30B—H30B	119.1
C30A—C31A—C32A	118.3 (4)	C30B—C31B—C32B	118.9 (4)
C30A—C31A—H31A	120.8	C30B—C31B—H31B	120.5
C32A—C31A—H31A	120.8	C32B—C31B—H31B	120.5
C23A—C32A—C31A	122.6 (4)	C23B—C32B—C31B	121.8 (4)
C23A—C32A—C33A	108.8 (3)	C23B—C32B—C33B	108.4 (3)
C31A—C32A—C33A	128.6 (4)	C31B—C32B—C33B	129.8 (4)
C42A—C33A—C34A	123.3 (4)	C42B—C33B—C34B	122.9 (4)
C42A—C33A—C32A	108.6 (3)	C42B—C33B—C32B	109.5 (3)
C34A—C33A—C32A	128.0 (3)	C34B—C33B—C32B	127.6 (4)
C35A—C34A—C33A	118.4 (3)	C35B—C34B—C33B	118.8 (4)
C35A—C34A—H34A	120.8	C35B—C34B—H34B	120.6
C33A—C34A—H34A	120.8	C33B—C34B—H34B	120.6
C34A—C35A—C36A	121.3 (4)	C34B—C35B—C36B	121.0 (4)
C34A—C35A—H35A	119.4	C34B—C35B—H35B	119.5
C36A—C35A—H35A	119.4	C36B—C35B—H35B	119.5
C37A—C36A—C35A	119.5 (4)	C37B—C36B—C35B	120.0 (4)
C37A—C36A—C41A	119.4 (4)	C37B—C36B—C41B	119.0 (4)
C35A—C36A—C41A	121.0 (4)	C35B—C36B—C41B	121.0 (4)
C38A—C37A—C36A	121.3 (4)	C38B—C37B—C36B	122.0 (4)
C38A—C37A—H37A	119.3	C38B—C37B—H37B	119.0
C36A—C37A—H37A	119.3	C36B—C37B—H37B	119.0
C37A—C38A—C39A	119.2 (4)	C37B—C38B—C39B	118.8 (4)
C37A—C38A—H38A	120.4	C37B—C38B—H38B	120.6
C39A—C38A—H38A	120.4	C39B—C38B—H38B	120.6
C40A—C39A—C38A	121.3 (4)	C40B—C39B—C38B	121.7 (4)
C40A—C39A—H39A	119.3	C40B—C39B—H39B	119.1
C38A—C39A—H39A	119.3	C38B—C39B—H39B	119.1
C39A—C40A—C41A	121.5 (4)	C39B—C40B—C41B	121.1 (4)
C39A—C40A—H40A	119.3	C39B—C40B—H40B	119.5
C41A—C40A—H40A	119.3	C41B—C40B—H40B	119.5
C40A—C41A—C36A	117.2 (3)	C40B—C41B—C36B	117.4 (3)
C40A—C41A—C42A	125.8 (3)	C40B—C41B—C42B	125.3 (3)
C36A—C41A—C42A	116.9 (3)	C36B—C41B—C42B	117.2 (3)
C33A—C42A—C41A	118.7 (3)	C33B—C42B—C41B	118.7 (3)
C33A—C42A—C22A	108.0 (3)	C33B—C42B—C22B	107.5 (3)
C41A—C42A—C22A	132.2 (3)	C41B—C42B—C22B	132.8 (3)
			
C22A—C1A—C2A—C11A	172.4 (3)	C22B—C1B—C2B—C11B	−174.9 (4)
C21A—C1A—C2A—C11A	−4.0 (4)	C21B—C1B—C2B—C11B	3.4 (4)
C22A—C1A—C2A—C3A	−20.4 (6)	C22B—C1B—C2B—C3B	17.9 (6)
C21A—C1A—C2A—C3A	163.2 (4)	C21B—C1B—C2B—C3B	−163.8 (4)
C11A—C2A—C3A—C4A	168.4 (4)	C11B—C2B—C3B—C4B	−165.3 (4)
C1A—C2A—C3A—C4A	2.3 (7)	C1B—C2B—C3B—C4B	0.8 (6)
C11A—C2A—C3A—C8A	−7.3 (6)	C11B—C2B—C3B—C8B	10.9 (5)
C1A—C2A—C3A—C8A	−173.4 (4)	C1B—C2B—C3B—C8B	176.9 (4)
C2A—C3A—C4A—C5A	−177.7 (4)	C8B—C3B—C4B—C5B	0.8 (6)
C8A—C3A—C4A—C5A	−2.0 (6)	C2B—C3B—C4B—C5B	176.9 (4)
C3A—C4A—C5A—C6A	0.2 (6)	C3B—C4B—C5B—C6B	1.8 (6)
C4A—C5A—C6A—C7A	0.5 (6)	C4B—C5B—C6B—C7B	−1.5 (7)
C5A—C6A—C7A—C8A	0.7 (7)	C5B—C6B—C7B—C8B	−1.4 (7)
C6A—C7A—C8A—C9A	174.8 (4)	C6B—C7B—C8B—C9B	−172.7 (4)
C6A—C7A—C8A—C3A	−2.5 (7)	C6B—C7B—C8B—C3B	4.0 (6)
C2A—C3A—C8A—C9A	1.8 (6)	C4B—C3B—C8B—C7B	−3.6 (6)
C4A—C3A—C8A—C9A	−174.2 (4)	C2B—C3B—C8B—C7B	179.9 (4)
C2A—C3A—C8A—C7A	179.1 (4)	C4B—C3B—C8B—C9B	173.1 (4)
C4A—C3A—C8A—C7A	3.1 (6)	C2B—C3B—C8B—C9B	−3.4 (5)
C7A—C8A—C9A—C10A	−173.4 (5)	C7B—C8B—C9B—C10B	172.3 (4)
C3A—C8A—C9A—C10A	3.9 (7)	C3B—C8B—C9B—C10B	−4.3 (6)
C8A—C9A—C10A—C11A	−3.9 (7)	C8B—C9B—C10B—C11B	4.3 (7)
C3A—C2A—C11A—C10A	7.6 (6)	C3B—C2B—C11B—C10B	−11.3 (6)
C1A—C2A—C11A—C10A	176.5 (4)	C1B—C2B—C11B—C10B	180.0 (4)
C3A—C2A—C11A—C12A	−168.2 (4)	C3B—C2B—C11B—C12B	167.0 (3)
C1A—C2A—C11A—C12A	0.7 (4)	C1B—C2B—C11B—C12B	−1.8 (4)
C9A—C10A—C11A—C2A	−1.9 (6)	C9B—C10B—C11B—C2B	3.5 (6)
C9A—C10A—C11A—C12A	173.0 (4)	C9B—C10B—C11B—C12B	−174.3 (4)
C2A—C11A—C12A—C13A	−175.7 (4)	C2B—C11B—C12B—C13B	179.1 (4)
C10A—C11A—C12A—C13A	8.8 (7)	C10B—C11B—C12B—C13B	−2.8 (7)
C2A—C11A—C12A—C21A	3.1 (5)	C2B—C11B—C12B—C21B	−0.6 (5)
C10A—C11A—C12A—C21A	−172.3 (4)	C10B—C11B—C12B—C21B	177.4 (4)
C21A—C12A—C13A—C14A	0.5 (7)	C21B—C12B—C13B—C14B	0.6 (7)
C11A—C12A—C13A—C14A	179.2 (4)	C11B—C12B—C13B—C14B	−179.2 (4)
C12A—C13A—C14A—C15A	−1.6 (7)	C12B—C13B—C14B—C15B	3.3 (7)
C13A—C14A—C15A—C16A	−178.5 (4)	C13B—C14B—C15B—C16B	175.3 (5)
C13A—C14A—C15A—C20A	−0.3 (7)	C13B—C14B—C15B—C20B	−1.4 (7)
C14A—C15A—C16A—C17A	175.2 (4)	C14B—C15B—C16B—C17B	−175.5 (5)
C20A—C15A—C16A—C17A	−2.9 (7)	C20B—C15B—C16B—C17B	1.3 (7)
C15A—C16A—C17A—C18A	−0.2 (7)	C15B—C16B—C17B—C18B	0.5 (7)
C16A—C17A—C18A—C19A	1.7 (7)	C16B—C17B—C18B—C19B	−0.8 (6)
C17A—C18A—C19A—C20A	0.0 (6)	C17B—C18B—C19B—C20B	−0.7 (6)
C18A—C19A—C20A—C15A	−3.1 (6)	C18B—C19B—C20B—C15B	2.5 (6)
C18A—C19A—C20A—C21A	−179.8 (4)	C18B—C19B—C20B—C21B	−179.6 (4)
C16A—C15A—C20A—C19A	4.5 (6)	C14B—C15B—C20B—C19B	174.0 (4)
C14A—C15A—C20A—C19A	−173.7 (4)	C16B—C15B—C20B—C19B	−2.7 (6)
C16A—C15A—C20A—C21A	−178.6 (4)	C14B—C15B—C20B—C21B	−4.0 (6)
C14A—C15A—C20A—C21A	3.3 (6)	C16B—C15B—C20B—C21B	179.2 (4)
C13A—C12A—C21A—C20A	2.5 (6)	C13B—C12B—C21B—C20B	−6.2 (6)
C11A—C12A—C21A—C20A	−176.4 (3)	C11B—C12B—C21B—C20B	173.6 (3)
C13A—C12A—C21A—C1A	173.4 (4)	C13B—C12B—C21B—C1B	−177.0 (4)
C11A—C12A—C21A—C1A	−5.6 (4)	C11B—C12B—C21B—C1B	2.8 (4)
C19A—C20A—C21A—C12A	172.4 (4)	C19B—C20B—C21B—C12B	−170.2 (4)
C15A—C20A—C21A—C12A	−4.3 (5)	C15B—C20B—C21B—C12B	7.7 (6)
C19A—C20A—C21A—C1A	4.5 (7)	C19B—C20B—C21B—C1B	−2.3 (7)
C15A—C20A—C21A—C1A	−172.2 (4)	C15B—C20B—C21B—C1B	175.6 (4)
C22A—C1A—C21A—C12A	−170.4 (4)	C22B—C1B—C21B—C12B	174.5 (4)
C2A—C1A—C21A—C12A	5.9 (4)	C2B—C1B—C21B—C12B	−3.8 (4)
C22A—C1A—C21A—C20A	−1.5 (7)	C22B—C1B—C21B—C20B	5.5 (7)
C2A—C1A—C21A—C20A	174.8 (4)	C2B—C1B—C21B—C20B	−172.8 (4)
C2A—C1A—C22A—C23A	131.8 (4)	C2B—C1B—C22B—C23B	−134.6 (4)
C21A—C1A—C22A—C23A	−52.6 (6)	C21B—C1B—C22B—C23B	47.4 (6)
C2A—C1A—C22A—C42A	−48.7 (6)	C2B—C1B—C22B—C42B	50.0 (6)
C21A—C1A—C22A—C42A	127.0 (4)	C21B—C1B—C22B—C42B	−128.0 (4)
C1A—C22A—C23A—C32A	178.2 (4)	C1B—C22B—C23B—C32B	−174.4 (4)
C42A—C22A—C23A—C32A	−1.4 (4)	C42B—C22B—C23B—C32B	1.8 (4)
C1A—C22A—C23A—C24A	−11.9 (6)	C1B—C22B—C23B—C24B	15.6 (7)
C42A—C22A—C23A—C24A	168.5 (4)	C42B—C22B—C23B—C24B	−168.1 (4)
C32A—C23A—C24A—C25A	169.8 (4)	C32B—C23B—C24B—C25B	−169.6 (4)
C22A—C23A—C24A—C25A	0.9 (7)	C22B—C23B—C24B—C25B	−0.5 (6)
C32A—C23A—C24A—C29A	−8.8 (5)	C32B—C23B—C24B—C29B	8.7 (5)
C22A—C23A—C24A—C29A	−177.7 (4)	C22B—C23B—C24B—C29B	177.8 (4)
C29A—C24A—C25A—C26A	−4.0 (6)	C29B—C24B—C25B—C26B	2.6 (6)
C23A—C24A—C25A—C26A	177.4 (4)	C23B—C24B—C25B—C26B	−179.1 (4)
C24A—C25A—C26A—C27A	−0.3 (7)	C24B—C25B—C26B—C27B	0.5 (6)
C25A—C26A—C27A—C28A	3.8 (7)	C25B—C26B—C27B—C28B	−3.3 (7)
C26A—C27A—C28A—C29A	−2.8 (7)	C26B—C27B—C28B—C29B	2.8 (7)
C27A—C28A—C29A—C30A	178.8 (4)	C27B—C28B—C29B—C30B	−178.1 (4)
C27A—C28A—C29A—C24A	−1.6 (6)	C27B—C28B—C29B—C24B	0.5 (7)
C25A—C24A—C29A—C30A	−175.5 (4)	C25B—C24B—C29B—C28B	−3.1 (6)
C23A—C24A—C29A—C30A	3.1 (6)	C23B—C24B—C29B—C28B	178.5 (4)
C25A—C24A—C29A—C28A	5.0 (6)	C25B—C24B—C29B—C30B	175.5 (4)
C23A—C24A—C29A—C28A	−176.4 (3)	C23B—C24B—C29B—C30B	−2.9 (5)
C28A—C29A—C30A—C31A	−177.7 (4)	C28B—C29B—C30B—C31B	175.9 (4)
C24A—C29A—C30A—C31A	2.8 (7)	C24B—C29B—C30B—C31B	−2.7 (6)
C29A—C30A—C31A—C32A	−3.1 (6)	C29B—C30B—C31B—C32B	2.4 (6)
C24A—C23A—C32A—C31A	9.0 (6)	C24B—C23B—C32B—C31B	−9.3 (6)
C22A—C23A—C32A—C31A	−179.8 (3)	C22B—C23B—C32B—C31B	179.4 (4)
C24A—C23A—C32A—C33A	−170.5 (3)	C24B—C23B—C32B—C33B	170.7 (3)
C22A—C23A—C32A—C33A	0.7 (4)	C22B—C23B—C32B—C33B	−0.6 (4)
C30A—C31A—C32A—C23A	−2.9 (6)	C30B—C31B—C32B—C23B	3.7 (6)
C30A—C31A—C32A—C33A	176.5 (4)	C30B—C31B—C32B—C33B	−176.3 (4)
C23A—C32A—C33A—C42A	0.3 (4)	C23B—C32B—C33B—C42B	−0.9 (5)
C31A—C32A—C33A—C42A	−179.2 (4)	C31B—C32B—C33B—C42B	179.1 (4)
C23A—C32A—C33A—C34A	−177.1 (4)	C23B—C32B—C33B—C34B	178.2 (4)
C31A—C32A—C33A—C34A	3.4 (7)	C31B—C32B—C33B—C34B	−1.8 (7)
C42A—C33A—C34A—C35A	−2.8 (6)	C42B—C33B—C34B—C35B	3.6 (6)
C32A—C33A—C34A—C35A	174.3 (4)	C32B—C33B—C34B—C35B	−175.4 (4)
C33A—C34A—C35A—C36A	−2.2 (6)	C33B—C34B—C35B—C36B	1.8 (6)
C34A—C35A—C36A—C37A	−174.9 (4)	C34B—C35B—C36B—C37B	174.9 (4)
C34A—C35A—C36A—C41A	2.8 (6)	C34B—C35B—C36B—C41B	−3.0 (6)
C35A—C36A—C37A—C38A	177.2 (4)	C35B—C36B—C37B—C38B	−178.3 (4)
C41A—C36A—C37A—C38A	−0.6 (6)	C41B—C36B—C37B—C38B	−0.4 (6)
C36A—C37A—C38A—C39A	−0.1 (6)	C36B—C37B—C38B—C39B	1.5 (6)
C37A—C38A—C39A—C40A	0.1 (6)	C37B—C38B—C39B—C40B	−1.6 (6)
C38A—C39A—C40A—C41A	0.7 (6)	C38B—C39B—C40B—C41B	0.5 (6)
C39A—C40A—C41A—C36A	−1.4 (6)	C39B—C40B—C41B—C36B	0.7 (6)
C39A—C40A—C41A—C42A	−179.0 (4)	C39B—C40B—C41B—C42B	178.6 (4)
C37A—C36A—C41A—C40A	1.3 (5)	C37B—C36B—C41B—C40B	−0.7 (5)
C35A—C36A—C41A—C40A	−176.4 (4)	C35B—C36B—C41B—C40B	177.1 (3)
C37A—C36A—C41A—C42A	179.1 (3)	C37B—C36B—C41B—C42B	−178.8 (3)
C35A—C36A—C41A—C42A	1.4 (5)	C35B—C36B—C41B—C42B	−1.0 (5)
C34A—C33A—C42A—C41A	7.0 (6)	C34B—C33B—C42B—C41B	−7.5 (6)
C32A—C33A—C42A—C41A	−170.6 (3)	C32B—C33B—C42B—C41B	171.6 (3)
C34A—C33A—C42A—C22A	176.4 (4)	C34B—C33B—C42B—C22B	−177.1 (4)
C32A—C33A—C42A—C22A	−1.2 (4)	C32B—C33B—C42B—C22B	2.1 (4)
C40A—C41A—C42A—C33A	171.6 (4)	C40B—C41B—C42B—C33B	−172.0 (4)
C36A—C41A—C42A—C33A	−6.0 (5)	C36B—C41B—C42B—C33B	6.0 (5)
C40A—C41A—C42A—C22A	5.3 (7)	C40B—C41B—C42B—C22B	−5.6 (7)
C36A—C41A—C42A—C22A	−172.3 (4)	C36B—C41B—C42B—C22B	172.4 (4)
C1A—C22A—C42A—C33A	−178.0 (4)	C1B—C22B—C42B—C33B	173.8 (4)
C23A—C22A—C42A—C33A	1.6 (4)	C23B—C22B—C42B—C33B	−2.4 (4)
C1A—C22A—C42A—C41A	−10.6 (7)	C1B—C22B—C42B—C41B	6.3 (7)
C23A—C22A—C42A—C41A	169.0 (4)	C23B—C22B—C42B—C41B	−169.9 (4)
